# Exploring the factors driving higher interactions in false amber necklace posts on Facebook

**DOI:** 10.1590/1807-3107bor-2024.vol38.0067

**Published:** 2024-08-05

**Authors:** Olívia Santana JORGE, Mariana Olímpio dos Santos REMIRO, Matheus LOTTO, Thiago CRUVINEL

**Affiliations:** (a) Universidade de São Paulo – USP, Bauru School of Dentistry, Department of Pediatric Dentistry, Orthodontics and Public Health, University of São Paulo, Bauru, SP, Brazil.

**Keywords:** Tooth Eruption, Communication, Telemedicine, Internet, Social Media

## Abstract

The consumption and sharing of information on social media contribute to the circulation of false health content. In this sense, popular belief-driven posts recommending the use of amber necklaces to prevent symptoms of teething can be easily found on social media, even when they offer health risks for children. Accordingly, this study aimed to characterize posts in Portuguese by identifying message-based factors associated with higher user interactions of Facebook. A total of 500 Facebook posts published in Portuguese between August 2016 and August 2021 were retrieved by CrowdTangle, and analyzed quantitatively according to time of publication, total user interaction, and post overperforming score, and then qualitatively by two independent investigators, according to motivation, authors’ profiles, and sentiment. The data were evaluated by descriptive analysis, Mann-Whitney U test, chi-square test, and multiple logistic regression models regarding interaction metrics. The posts were published predominantly by commercial profiles, with social, psychological, and financial motivations, and expressed positive sentiment. Furthermore, time of publication (OR=2.65), regular user profiles (OR=3.30), and neutral/negative sentiment (OR=2.76) were associated positively with total interactions, whereas only time of publication was associated with post overperforming scores (OR=1.65). In conclusion, Facebook posts promoting efficacy of amber necklaces in managing teething symptoms are primarily created by commercial profiles with vested interests in marketing the product. The key factors that contribute to the success of posts containing false information about amber necklaces on Facebook can be identified by considering the author’s profile, sentiment, and time of publication.

## Introduction

Facebook remains at the forefront of the global social media arena, and has become a key channel for disseminating health-related information among the population.^1,2^ Furthermore, it is recognized as one of the most significant platforms for engaging in content that aligns with existing user biases, often rife with misinformation.^3^ In Brazil, Facebook boasts a substantial user base, projected to reach about 127 million active users by 2024.^4^ Additionally, Brazil is identified as one of the countries where the population is highly susceptible to the perils of misinformation, according to the World Economic Forum (2024).^5^ Despite the widespread use of social media for accessing and sharing health-related information, there is a notable absence of scientific backing in much of this content, leading to the spread of falsehoods.^6-8^ This situation creates a paradox in which the consumption and dissemination of health information on platforms like Facebook can unintentionally expose users to misleading content. Such exposure has the potential to alter traditional health behaviors negatively, by encouraging the formation or strengthening of harmful beliefs. These, in turn, can significantly deteriorate the quality of health decisions made by users.^9^

One example of such false health content is that of the amber necklace, which is sold as an amulet promising relief from the symptoms of teething, based on the analgesic and anti-inflammatory effects of the succinic acid found in amber beads.^10^ Although teething is often reported as a stressful period for babies, there is scarce evidence linking specific symptoms to tooth eruption.^11^ Nevertheless, parents and caregivers seek solutions to relieve their baby’s discomfort. Previous studies have shown potential health risks of the amber necklace, such as allergic reactions, strangulation, and microbial proliferation.^12-14^ In vitro studies also indicate that the succinic acid concentration of Baltic amber is negligible, and that acid availability requires very high temperatures to release the effects, thus making the purported benefits of the amulet implausible.^10^

Despite the lack of evidence and potential health risks, the amber necklace is commonly sold and used, and increasing interest has been observed in online searches for the product.^15^ Social media platforms, including Facebook, play a significant role in disseminating health information, which can reinforce popular beliefs and contribute to the circulation of false content.^16,17^ Users within a network may adopt similar viewpoints, including erroneous ones, underscoring the critical need for digital strategies and tools to combat false health content on social media.^18^Notably, individuals who fall for a false health claim are likely to be more vulnerable to other types of misinformation.^19^ This pattern emphasizes the importance of addressing misinformation comprehensively to protect public health.

This study aims to characterize the factors associated with total user interactions with Facebook posts in Portuguese, containing false information on the efficacy of amber necklaces in preventing teething symptoms. Specifically, the study examined the authors’ profile, sentiment, motivation and audience to better understand how false health content spreads on social media.

## Methods

### Study design

This digital study showcased 500 Portuguese-language Facebook posts that contained false content about using amber necklaces to prevent the symptoms of teething. Initially, 684 amber necklace-related posts published between August 2016 and August 2021 were retrieved from CrowdTangle^TM^, and sorted by total user interaction. Subsequently, two independent investigators evaluated the posts qualitatively using content analysis for the author’s profile, sentiment, and motivation (OSJ and MOSR). Lastly, the data were evaluated by descriptive analysis, Mann-Whitney U test, chi-square test, and multiple logistic regression models regarding interaction metrics. See more details below.

### Ethics

This study was exempt from approval by the Human Research Ethics Committee of the Bauru School of Dentistry, because federal regulations do not apply to research using publicly available data that does not involve human subjects.

### Search strategy

The search strategy was structured meticulously using a combination of Portuguese terms and relevant hashtags pertaining to amber necklaces and primary teeth. The strategy used Boolean operators to enhance the retrieval of posts, by incorporating only terms proven to augment the search scope. The refined search strategy is outlined as follows: (#colardeambar OR #ambarbaltico OR “colar de âmbar” OR “âmbar báltico” OR “ambar baltico” OR “colar de ambar”) AND (dente OR dentinho OR dentes OR dentinhos).

### Data collection

This study used the CrowdTangle^TM^ web scraping technology to collect the posts. This tool by Meta Inc. performs online analysis and gains insights, and has been applied in previous social media-driven studies. It was given to our research group to investigate information disorders in dentistry. CrowdTangle^TM^ provides various metrics on Facebook for a specific topic based on keywords, such as the number of posts, publication date (day and time), profile information (name and country), type of post, post contents, total interaction (sum of the number of post likes, comments, and views), and overperforming score.

The overperforming score indicates the performance of a post in terms of actual interaction divided by expected interaction, based on the number of followers of the author’s profile. Positive scores indicate good performance posts, where more users were reached than the number of the author’s friends, while negative scores indicate the opposite. CrowdTangle^TM^ generates benchmarks to identify expected values using the last 100 posts from a given account. The algorithm eliminates the top and bottom 25% of the posts, and calculates the mean number of interactions, considering the middle 50% of the posts at different time intervals (15 minutes old, 60 minutes old, 5 hours old, etc.). Subsequently, when the account in question publishes a new post, the platform compares the post metrics with the calculated average, and multiplies the difference by the weights on each dashboard.^20-22^

On September 8, 2021, CrowdTangle^TM^ was used to search for Portuguese Facebook posts published between August 2016 and August 2021, excluding videos. A dataset with information on 684 posts was downloaded in .csv format including the post ID, publication date and time, profile information (name and country), type of post, post content, total interaction (the sum of post likes, comments, and views), and the overperforming score. The posts were ordered by total interaction.

### Pre-processing dataset

The eligibility of a sample of the 500 most interacted posts for the study was confirmed by a single investigator (OSJ), who carefully reviewed all the available links, and read through 650 posts in full. This rigorous approach ensured the reliability and accuracy of our findings.

Duplicates (n = 38) and posts containing accurate information on the topic (n = 112) were excluded prior to the qualitative analysis (Figure). The remaining posts were then printed, and anonymized by blacking out names, profiles, and people’s eyes in images. Standardization was ensured, and inconsistencies were prevented by numbering the posts, and saving them in sequence on a Google Slides file (Google, Mountain View, USA), later converted to a .pdf file. This systematic process provided ethical analysis of messages by different investigators at different times.

**Figure:**
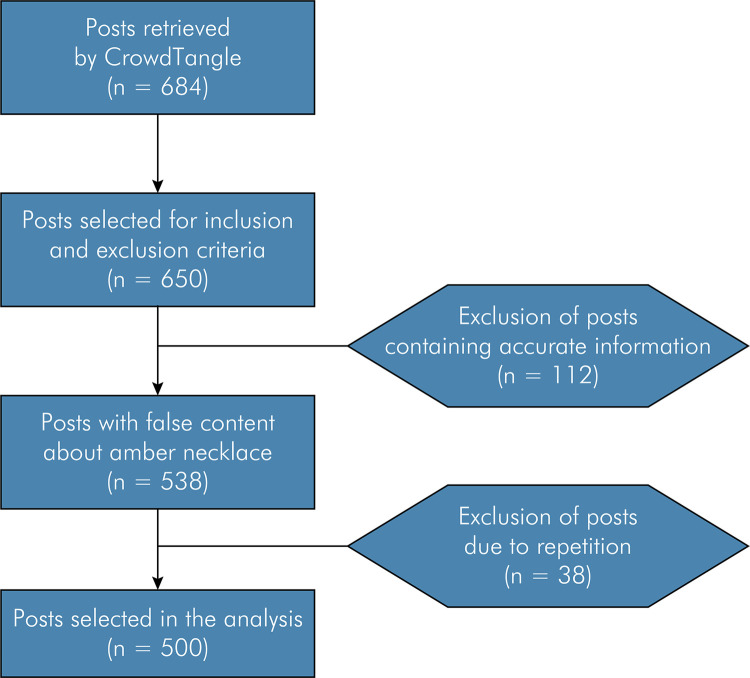
Flowchart of the selection of posts for analysis.

### Qualitative analysis

Two trained and calibrated investigators (OSJ and MOSR) analyzed the posts independently using three criteria: motivation, author’s profile, and sentiment, yielding absolute inter-examiner agreement values that ranged from 0.84 to 0.95. The training process consisted of reviewing several publications on the amber necklace, together with a third investigator (TC), for the purpose of discussion and establishing the criteria for evaluation. Afterwards, 10% of the sample (50 posts) was evaluated until the desired level of interexaminer agreement was obtained (greater than 0.8). This process was performed three times.

Motivations for posting false content on the internet were grouped into several categories, i.e., social (to create a connection with a particular social group), psychological (to reinforce or influence users with information, while seeking to gain some prestige), financial (to profit from misleading content used to advertise or sell a product), and political (to influence public opinion for endorsement of political positions), following the classification described by Wardle and Derakhshan.^23^

The authors’ profiles were classified based on their name and descriptions, including regular users (common users, celebrities, and public figures), health providers (names accompanied by the prefix “doctor” or users who reported activities related to medicine, dentistry or any healthcare activity), businesses (stores or companies), blogs/groups (communities, blogs or groups), news agencies (news channels or journalistic media), and NGOs (non-governmental organizations).

Post sentiments were classified as positive, neutral, or negative, based on their content and the accompanying emojis. Posts expressing admiration for the amber necklace or the supposed effects of the amulet and/or containing emojis linked to positive feelings were classified as expressing positive sentiment. Posts that provided information on the characteristics or benefits of the necklace, and that had no emojis were classified as expressing neutral sentiment. Posts that conveyed distress, fear, sadness, or other negative emotions related to possible symptoms of teething were classified as expressing negative sentiment.

The objectivity of our analysis was ensured by our classifying contents as false, if they mentioned any property, recommendation, or effect of the amber necklace regarding symptoms of teething with no scientific basis. Examples of falsehoods based on the lack of evidence found in the literature include: the release and absorption of succinic acid based on body temperature, and the analgesic action of succinic acid drawn from amber stones in relieving teething symptoms, as well as claims that the necklace guarantees safety against choking or swallowing of the beads. If there was any disagreement between the investigators, the posts were re-evaluated and discussed until a consensus was reached.

### Statistical analysis

The statistical analysis was performed using the IBM SPSS Statistics for Macintosh (v. 21.0; IBM Corp.; Armonk, USA). Examiner calibration was confirmed by determining the absolute interexaminer agreement using the intraclass correlation coefficient (ICC). Different variables were compared by dichotomizing them as follows: time of publication (≤ 1,224 days or > 1,224 days), motivation (non-financial or financial), authors’ profile (regular users or business), sentiment (negative/neutral or positive), type of publication (photo/status or link), total interaction (≤ 8 or > 8), and overperforming score (≤ -0.60 or > -0.60). The continuous variables were dichotomized by their median values. Dental offices, news agencies, and business profiles were grouped based on their financial background. The decision to dichotomize sentiment in this study was warranted based on previous research linking positive emotions to increased engagement rates of the social media user.^24^ This approach was taken to determine whether a similar pattern of results would emerge for false messages related to the amber necklace.

The distribution and consistency of the dataset was evaluated by applying the Kolmogorov-Smirnov test to examine normality, and the Levene test to assess homogeneity. Consistent with expectations, the distribution of data notably diverged from normality in the context of social media interaction metrics, which is often marked by significant variability across posts. A common observation is that there is an inverse relationship between the number of posts and their cumulative interactions. In light of the non-normal distribution of the data, the Mann-Whitney U test was applied subsequently to compare total interactions and overperforming scores among the dichotomized variable groups. Additionally, the chi-square test was employed to compare the distribution of motivation categories among the dichotomized variable groups.

Furthermore, multiple logistic regression models were developed to assess the association of overperforming scores and total interaction with distinct variables. Only factors with significant Wald statistics in the simple analyses were included in the multiple models. p-values < 0.05 were considered statistically significant in all the analyses.

## Results

Overall, the majority of posts analyzed in this study were created by business profiles (412, 82.4%) with financial motivations (455, 91.0%), and expressed positive sentiments (461, 92.2%) through photos/status (473, 94.6%). However, all posts also had social and psychological motivations.

Table 1 provides a comparison of the total interaction and overperforming scores for dichotomized variable groups. It was found that posts with non-financial motivations (*e.g., “We’re going through that annoying phase of teething, with six teeth all coming in at the same time. For the thousandth time, I’m trying the amber necklace, but thank God this phase will pass”*) negative/neutral sentiment (e.g., “*It has been three days since Bê broke her necklace, and I haven’t slept at all this whole time, and her teeth are finally coming through”*), regular user profiles, and more than 1,224 days since publication had significantly higher total interactions. Similarly, overperforming scores were significantly higher in posts with regular user profiles and more than 1,224 days since publication.

**Table 1:** Comparison of average (±SD) and median (IQR) total interaction and overperforming scores regarding time of publication, motivation, profile, sentiment, and type of publication (Mann-Whitney U test, p < 0.05). All sample (n = 500).

Variable	Total Interactions				Overperforming score			

	n (%)	Average (± SD)	Median (IQR)	p-value	n (%)	Average (± SD)	Median (IQR)	p-value
Days past of publication								
≤ 1,224 days	252 (50.4)	350 (± 2130)	6 (14)	< 0.001*	252 (50.4)	-0.45 (± 40.86)	-0.67 (0.74)	0.004*
> 1,224 days	248 (49.6)	168 (± 599)	13 (70)		248 (49.6)	1.11 (± 32.46)	-0.50 (2.30)	
Motivation								
Non-financial	45 (9.0)	215 (± 492)	36 (93)	< 0.001*	45 (9.0)	-4.62 (± 19.80)	-0.75 (4.28)	0.671
Financial	455 (91.0)	264 (± 1640)	7 (20)		455 (91.0)	0.81 (± 38.16)	-0.60 (1.64)	
Author’s profile								
Regular users	88 (17.6)	942 (± 3358)	58 (170)	< 0.001*	88 (17.6)	-6.60 (± 20.29)	-1.21 (8.45)	0.010*
Business	412 (82.4)	114 (± 698)	7 (16)		412 (82.4)	1.80 (± 39.42)	-0.56 (1.57)	
Sentiment								
Negative/Neutral	39 (7.8)	473 (± 1271)	36 (282)	< 0.001*	39 (7.8)	11.71 (± 62.85)	0.84 (5.57)	0.071
Positive	461 (92.2)	242 (± 1594)	7 (21)		461 (92.2)	-0.64 (± 33.75)	-0.60 (1.64)	
Type of publication								
Photo/Status	473 (94.6)	265 (± 1611)	8 (22)	0.075	473 (94.6)	0.59 (± 37.69)	-0.60 (1.69)	0.141
Link	27 (5.4)	161 (± 475)	25 (97)		27 (5.4)	-4.38 (± 18.18)	1.21 (2.63)	

Table 2 summarizes the distribution of motivation categories according to dichotomized variable groups, based on the presence or absence of financial motivation. The analysis showed a significantly higher frequency of financial motivation in posts that were created by business profiles, which expressed positive sentiment (*e.g., “Is there a tooth coming in there? I use it and love it!”*), and which included posts with photos/status (without a link).

**Table 2:** Distribution of motivation by total interaction, overperforming score, author’s profile, sentiment, time of publication, and type of publication (chi-square, p < 0.05). Different superscript lowercase letters indicate significant statistical differences between categories of motivation.

Variable	Motivation		X^2^	p-value

	Non-financial	Financial		

	n = 45	n = 455		
Total interaction				
≤ 8	8 (17.8%)^a^	251 (55.2%)^a^	22.926	< 0.001*
> 8	37 (82.2%)^b^	204 (44.8%)^b^		
Overperforming score				
≤ -0.6	23 (51.1%)^a^	232 (51.0%)^a^	< 0.001	0.988
> -0.6	22 (48.9%)^a^	223 (49.0%)^a^		
Author’s profile				
Regular users	42 (93.3%)^a^	46 (10.1%)^a^	195.571	< 0.001*
Business	3 (10.1%)^b^	409 (89.1%)^b^		
Sentiment				
Negative/Neutral	20 (44.4%)^a^	19 (4.2%)^a^	92.334	< 0.001*
Positive	25 (55.6%)^b^	436 (95.8%)^b^		
Time of publication				
≤ 1,224 days	17 (37.8%)^a^	235 (51.6%)^a^	3.152	0.076
> 1,224 days	28 (62.2%)^a^	220 (48.4%)^a^		
Type of publication				
Photo/Status	34 (75.6%)^a^	439 (96.5%)^a^	35.109	< 0.001*
Link	11 (24.4%)^b^	16 (3.5%)^b^		

The multiple logistic regression models for total interaction and overperforming scores have been presented in Table 3. The analysis revealed that regular users (OR = 3.297; p < 0.001), older posts (OR = 2.648; p < 0.001), and negative/neutral sentiment (OR = 2.758; p = 0.022) were associated positively with higher total user interactions, while older posts were associated positively with higher overperforming scores (OR = 1.646; p = 0.006) (Table 3).

**Table 3:** Multiple logistic regression models of total interaction and overperforming score.

Variable	B^a^	S.E.^b^	Wald	p-value	OR^c^	95%IC	

						LCI	UCI
Total interaction (> 8)							
Author’s profile (Regular users)	1.193	0.336	12.591	< 0.001	3.297	1.706	6.372
Time of publication (> 1,224)	0.974	0.193	25.521	< 0.001	2.648	1.815	3.864
Motivation (Non-financial)	0.338	0.517	0.428	0.513	1.403	0.509	3.865
Sentiment (Negative/Neutral)	1.014	0.442	5.268	0.022	2.758	1.160	6.558
Constant (y-intercept)	-0.849	0.146	33.909	< 0.001	0.428		
Overperforming score (> -0.60)							
Time of publication (> 1,224)	0.498	0.180	7.632	0.006	1.646	1.156	2.343
Constant (y-intercept)	-0.288	0.127	5.108	0.024	0.024		

^a^Unstandardized coefficient; ^b^Standard error; ^c^Odds ratio.

## Discussion

The findings of the present study showed that the majority of amber necklace-related posts were created by business profiles with social, psychological, and financial motivations, and expressed positive sentiment. Moreover, the time of publication, regular user profiles, and neutral/negative sentiment were associated positively with total interactions. Moreover, the time of publication was related positively to the overperforming scores.

These findings are consistent with a previous study that also characterized Facebook posts for amber teething necklaces, albeit conducted in English^25^. In this prior study, it was also found that the majority of posts were made by business profiles, notable for expressing positive sentiments driven by social, psychological, and financial motivation. In addition, a positive association was observed between the posting time and both higher user interactions and overperformance scores.

The positive association of time of publication with total interaction and overperforming score can be explained by the extended availability of posts on social media.^20^ Facebook content can be actively accessed over time according to user interests, and the older posts could have higher overperforming scores due to the intense spread of the new content some years ago, as explained by the innovation diffusion curve.^15,26,27^

We had expected most of the posts about amber necklaces to come from stores with a financial interest. This is because amber necklaces are marketed primarily as accessories for babies, and the main target audience is parents and caregivers.^28^ Although the study did not aim to distinguish between different types of information disorders, some theories suggest that financial motivation can be a direct indicator of the intent behind a message.^29^ Therefore, analyzing the digital content related to amber necklaces on social media could serve as a useful model for gaining insights into the phenomenon of disinformation in dental issues.

The prevalence of positive sentiment was also expected, since companies use positive devices to arouse the consumer’s desire to buy, like speeches or emojis related to positivity, prizes, and discounts.^30,31^ Furthermore, images are crucial for online product sales, since consumers cannot physically touch or inspect the product.^28^ Therefore, it was no surprise that most posts promoting the amber necklace featured images. Facebook itself is a visually-oriented social media platform, thus making it even more important for businesses to use eye-catching visuals in their posts. As a result, business profiles dominated the promotion of the amber necklace, which used effective communication and sales strategies, along with flashy images, to evoke positive emotions and entice the consumer to purchase the product.

On the other hand, posts published by regular user profiles were related to significantly higher total interaction. Celebrities and digital influencers have great persuasive power over consumers for branding recommendations in social media.^32^ For example, a previous study demonstrated that a picture on Instagram of top model Gisele Bündchen holding her daughter, who was wearing an amber necklace, significantly influenced the list of top queries in Brazil related to this item of jewelry.^15^

In addition, ordinary users, like concerned parents and caregivers, create digital environments of interaction between individuals with similar health experiences. Individuals with a greater need for belonging can be more easily persuaded by this kind of Facebook post when digital influencers and celebrities endorse false content about the amber necklace.^33-35^ Interestingly, posts that expressed neutral/negative sentiment were significantly related to higher total interaction, even though most posts had a positive sentiment. In this context, sellers who emphasized distress and the negative symptoms associated with teething may have evoked a feeling of understanding and acceptance from their target audience, by presenting the amber necklace as a possible solution. The display of emotional support and empathy in the digital environment can enhance user confidence and make users more receptive to sales strategies, ultimately leading to increased trust in the company.^36,37^

These findings can significantly enhance the understanding of the dynamics surrounding unreliable content related to amber necklaces, particularly in terms of their ability to capture the attention of social media users and spread widely. In this regard, it is noteworthy that posts created by regular users and those with a negative or neutral sentiment tend to garner more likes and shares compared to their alternatives. Additionally, these results can inform educational approaches that help social media users access reliable digital information regarding children’s oral health. Furthermore, policymakers must acknowledge the adverse impact of false posts on amber necklaces and other dental issues, since they pose a risk of fatal injury like strangulation and aspiration. The proliferation of confirmation bias further compounds this problem.^35,38^ As such, dental professionals must be aware of the popularity of amber necklaces and prioritize child-centered care by using effective and individualized educational strategies. Dental professionals can promote better oral health outcomes for children by respectfully addressing parental doubts and gradually changing negative beliefs.^34^

This study had some limitations. First, the investigators were unable to determine the authors’ intentionality precisely, hence leading them to assume the interpretation of results using a definition of misinformation as an umbrella concept.^29^ It is worth highlighting that this constraint is a recurring issue in numerous studies on information disorder, since it is exceedingly challenging to verify an unspoken third-person’s intention objectively and precisely.^39,40^ Second, the sample size of our study was limited due to the challenges inherent in conducting human-based content analysis, considering the practicality of labeling datasets manually, thus consistent with the approach used in prior studies.^20,41^ Third, CrowdTangle exclusively tracks accounts deemed influential, such as those with verified profiles (50,000+ followers) and public groups with over 95,000 likes. While it is not feasible to collect every post from ordinary users, the majority of interactions with content occur within the subset of posts included in our sample. Therefore, the analysis focused on posts that truly engendered significant engagement. Lastly, our findings were based solely on posts written in Portuguese. The cultural aspects specific to this language and to different regions may have influenced the factors associated with the dissemination of false information about amber necklaces. Further research is needed to assess whether similar trends exist in other languages and cultures.

## Conclusion

In summary, it is evident that Facebook posts promoting the efficacy of amber necklaces in managing the symptoms of teething are primarily created by commercial profiles with vested interests in marketing the product. Interestingly, older posts and those published by regular users with neutral or negative sentiments tended to receive more shares and comments, thus leading to increased popularity. Furthermore, higher user interaction rates were observed in posts published earlier.

Future investigations are inherently capable of elucidating the ramifications of the amber necklace discourse across diverse social media platforms. Furthermore, these inquiries can shed light on the consequences of engaging with oral health-related information on Facebook, specifically with regard to its influence on the health behaviors exhibited within the population.
